# The Benefit of Reactivating p53 under MAPK Inhibition on the Efficacy of Radiotherapy in Melanoma

**DOI:** 10.3390/cancers11081093

**Published:** 2019-08-01

**Authors:** Mohammad Krayem, Malak Sabbah, Ahmad Najem, An Wouters, Filip Lardon, Stephane Simon, François Sales, Fabrice Journe, Ahmad Awada, Ghanem E. Ghanem, Dirk Van Gestel

**Affiliations:** 1 Laboratory of Oncology and Experimental Surgery, Institut Jules Bordet, Université Libre de Bruxelles, Rue Héger-Bordet 1, 1000 Brussels, Belgium; 2 Department of Radiation Oncology, Institut Jules Bordet, Université libre de Bruxelles, 1000 Brussels, Belgium; 3 Center for Oncological Research (CORE), University of Antwerp, 2610 Wilrijk, Belgium; 4 Department of Human Anatomy and Experimental Oncology, Université de Mons (UMons), Research Institute for Health Sciences and Technology, 7000 Mons, Belgium; 5 Department of Internal Medicine, Institut Jules Bordet, Université Libre de Bruxelles, 1000 Brussels, Belgium

**Keywords:** radiotherapy, ^V600E^BRAF inhibition, p53 activation, intrinsic and acquired resistance, melanoma

## Abstract

Radiotherapy (RT) in patients with melanoma historically showed suboptimal results, because the disease is often radioresistant due to various mechanisms such as scavenging free radicals by thiols, pigmentary machinery, or enhanced DNA repair. However, radiotherapy has been utilized as adjuvant therapy after the complete excision of primary melanoma and lymph nodes to reduce the rate of nodal recurrences in high-risk patients. The resistance of melanoma cells to radiotherapy may also be in relation with the constitutive activation of the MAPK pathway and/or with the inactivation of p53 observed in about 90% of melanomas. In this study, we aimed to assess the potential benefit of adding RT to BRAF-mutated melanoma cells under a combined p53 reactivation and MAPK inhibition in vitro and in a preclinical animal model. We found that the combination of BRAF inhibition (vemurafenib, which completely shuts down the MAPK pathway), together with p53 reactivation (PRIMA-1^Met^) significantly enhanced the radiosensitivity of BRAF-mutant melanoma cells. This was accompanied by an increase in both p53 expression and activity. Of note, we found that radiation alone markedly promoted both ERK and AKT phosphorylation, thus contributing to radioresistance. The combination of vemurafenib and PRIMA-1^Met^ caused the inactivation of both MAPK kinase and PI3K/AKT pathways. Furthermore, when combined with radiotherapy, it was able to significantly enhance melanoma cell radiosensitivity. Interestingly, in nude mice bearing melanoma xenografts, the latter triple combination had not only a synergistic effect on tumor growth inhibition, but also a potent control on tumor regrowth in all animals after finishing the triple combination therapy. RT alone had only a weak effect. In conclusion, we provide a basis for a strategy that may overcome the radioresistance of BRAF-mutated melanoma cells to radiotherapy. Whether this will translate into a rational to use radiotherapy in the curative setting in BRAF-mutated melanoma patients deserves consideration.

## 1. Introduction

Over the past 10 years, increased biological understanding and access to innovative therapeutic substances have transformed advanced melanoma into a new oncological model for treating solid cancers. Treatments that target B-Raf proto-oncogene serine/threonine kinase (BRAF) V600 (Val600) mutations using selected BRAF inhibitors combined with mitogen-activated protein kinase (MAPK) inhibitors have significantly improved response and overall survival [1]. Furthermore, advanced cutaneous melanoma has developed into a prototype for testing checkpoint-modulating agents, which has increased hope for long-term tumor containment and a potential cure [2]. The next breakthrough in cancer treatment following the success in immunotherapy and using immune checkpoint inhibitors is the oncolytic virus therapy, which has been approved recently by the European Medicines Agency (EMA) and the United States (US) Food and Drug Administration (FDA) [3,4,5,6].

On the other hand, melanoma is commonly regarded as a radioresistant tumor entity. However, radiotherapy (RT) is used as adjuvant treatment for patients suffering from advanced nodal disease, reducing the risk of regional tumor relapse [7,8]. Several clinical studies, including a randomized phase III multicenter clinical trial, indeed showed that postoperative radiation therapy significantly reduces the risk of lymph node recurrence in patients who have undergone therapeutic lymphadenectomy, yet leaving overall survival dissatisfyingly unchanged [8,9]. More importantly, RT can provide effective palliation for the 40% to 50% of patients with unresectable or metastatic disease that produces bone pain, epidural spinal cord compression, or central nervous system dysfunction [7,8]. Emerging data have challenged this viewpoint, and RT is now considered an effective treatment option in some settings [10,11,12], although its use has dwindled in recent years with the advent of successful new therapies and several FDA-approved immunotherapies options for the competing risk of melanoma systemic disease [7]. 

Cancer radiosensitizers are promising agents that enhance injury to tumor tissue by accelerating DNA damage and producing free radicals [13]. Several strategies to improve the therapeutic ratio are currently under investigation to enhance the radiation effect, thereby preventing tumor recurrence or progression [13,14,15,16]. In melanoma, there is a compelling rationale to identify promising molecular targeting agents that may sensitize melanoma to radiation. Melanoma resistance to radiotherapy may be due to constitutive activation of MAPK pathway signaling. Among these, activating BRAF mutations occurs in 50% to 60% of melanomas [17]. These mutations opened new therapeutic perspectives, targeting the MAPK pathway with BRAF and MEK inhibitors [18]. Nevertheless, not all patients respond to these agents; a minority of patients’ present primary resistance (intrinsic resistance), while all patients develop secondary resistance (acquired resistance) [19,20,21]. However, MAPK inhibition potentiates the effect of radiotherapy in melanoma [11,22,23].

A second main cause of cancer radioresistance is the inactivation of p53 [24,25,26], as wild-type p53 plays a prominent role in the radiosensitization of cancer cells [27,28,29]. In melanoma, the p53 gene is rarely mutated (5–17%) but the wild-type form is frequently inactivated [30,31,32,33]. 

p53 is inactivated in melanoma by a variety of mechanisms, of which overexpression of MDM2 (mouse double minute 2). However, the MDM2 inhibitor Nutlin-3 causes only modest p53-mediated cell death in melanoma [34,35,36]. Furthermore, phosphorylated nuclear iASPP (Inhibitor of apoptosis-stimulating protein of p53) has been reported to correlate with MDM2 overexpression in wild-type p53 melanoma cells [37], highlighting the need to co-target, at least, MDM2 and iASPP to optimally reactivate p53. Finally, the upregulation of MDM4 expression is one of the key mechanisms of p53 inactivation in melanoma [32]. MDM4 overexpression renders most primary melanoma cultures relatively immune to specific MDM2 inhibition [32]. However, MDM4 was found weakly expressed in mutant BRAF vemurafenib-resistant melanoma cell lines [36]. Thus, other mechanisms causing p53 inactivation may be at play in these cell lines. Aberrant expression of additional p53 co-factors (directly binding p53) and regulators (modulating p53 activity) has been described in melanoma, suggesting possible roles in inactivating melanoma p53. These include PIASy (protein inhibitor of activated STAT), the histone acetyl transferase Tip60 (HTATIP), Y boxbinding protein 1, p63, and p73 [38]. Therefore, the inhibitory mechanism or mutational status directs p53 reactivation and emerges as a broad spectrum and promising therapeutic strategy. Such possibility exists thanks to a few p53-binding molecules that not only rescue mutant p53 but also activate the function of wild-type p53 by affecting its conformation e.g., p53 Reactivation and Induction of Massive Apoptosis (PRIMA)-1Met [39]. Interestingly, most p53 activators selectively target the ability of only one of the regulators to interact with p53, leaving the other free to operate. Thus, mutated or inactivated p53 may represent a complementary therapeutic target for melanoma. 

Many reports showed a p53-dependent radiosensitivity [40,41]. From this p53 dependency, it is important that radiosensitizers act on cancer cells regardless of p53 status. PRIMA-1 is a small molecule that has the ability to convert mutant and wild-type inactive p53 to an active conformation, restoring DNA binding and transcriptional activity. PRIMA-1 and its methylated form PRIMA-1^Met^ (APR-246), a non-genotoxic candidate drug for mutant p53 reactivation, have been shown to induce p53-mediated apoptosis and cell cycle arrest in different types of cancer as well as be potent inducers of oxidative stress [39]. The safety of APR-246/PRIMA-1^Met^ has been tested in a phase I clinical trial published in 2012 [42]. More recently, we evaluated the therapeutic impact of combining BRAF inhibition with the direct pharmacological reactivation of p53 [36]. Strikingly and similarly, we also found that direct p53 reactivation (PRIMA-1^Met^, APR-246) brakes resistance and synergizes with MEK inhibition to induce massive apoptosis in NRAS (Neuroblastoma rat sarcoma (RAS) viral oncogene homolog) -mutant melanoma cells with wild-type or mutant p53 [43]. 

However, the potential benefit of reactivating p53 in combination with MAPK inhibitors on the efficacy of RT in melanoma has not been explored. Moreover, the resistance of melanoma cells to RT may very well be in relation with the constitutive activation of MAPK pathway (including RTK, NRAS, and BRAF mutations) and/or with the inactivation of p53 observed in about 90% of melanomas [31,33]. In order to clarify this question, we evaluated in vitro and in vivo the effect of combining reactivation of p53 with MAPK inhibition on the efficacy of RT in BRAF-mutated melanoma with intrinsic and acquired resistance to BRAF inhibitors. 

## 2. Results

### 2.1. p53 Activator (PRIMA-1^Met^) Improves the Radiosensitizing Effect of BRAF Inhibitor (Vemurafenib) in ^V600E^BRAF Mutant Melanoma Cells 

**Figure 1:**
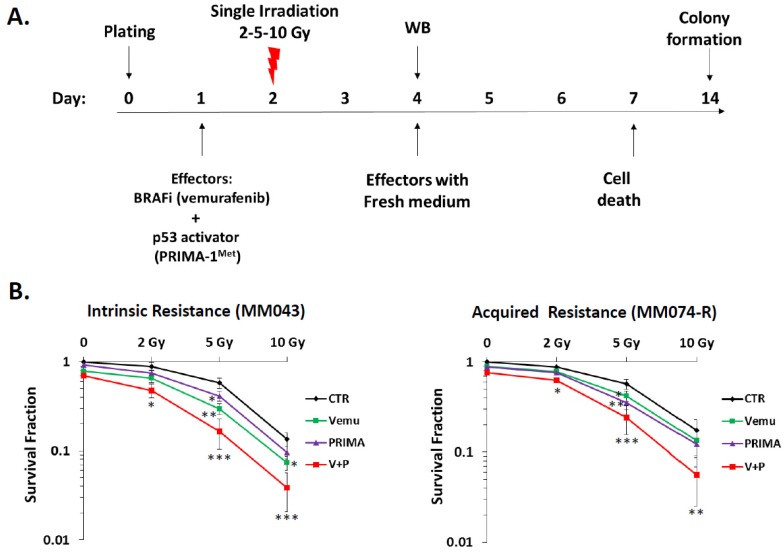

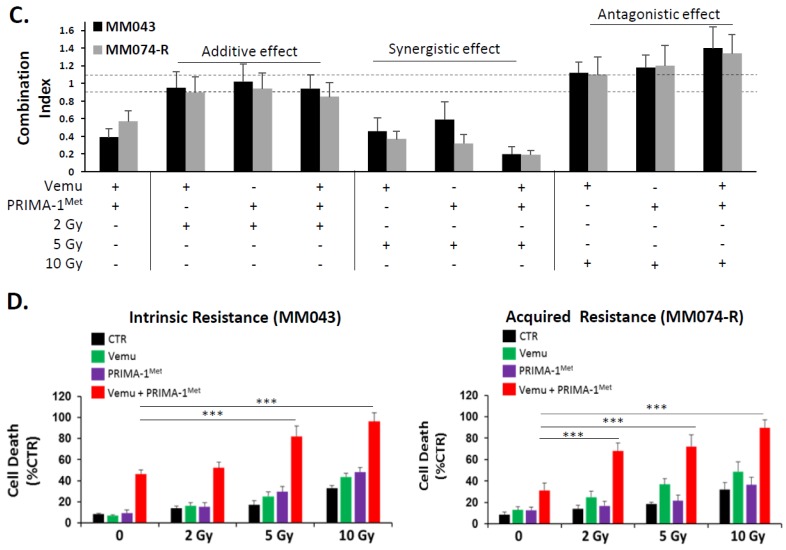
Combining p53 activation and B-Raf proto-oncogene serine/threonine kinase (BRAF) inhibition sensitizes melanoma BRAF mutant melanoma cells to irradiation. (**A**) In the workflow, cells were cultured on day 0, effectors were added on day 1, and radiotherapy (RT) was done on day 2. Western blot (WB) analysis followed two days after RT (day 4). Cell death and colony formation were evaluated on days 7 and 14, respectively. Effectors with fresh medium were changed every three days. (**B**) Clonogenic survival assay of human melanoma cell lines with intrinsic resistance (MM043) and acquired resistance (MM074-R) to vemurafenib with different irradiation doses (2 Gy, 5 Gy, and 10 Gy) alone or in combination with vemurafenib (Vemu, 0.1 µM) and/or PRIMA-1^Met^ (PRIMA-1^Met^, 20 µM). Surviving fractions were calculated relative to plating efficiencies. Data were presented as the mean ± standard error of at least three independent experiments. Gy, Gray; CTR: untreated control; Vemu, vemurafenib; p53 Reactivation and Induction of Massive Apoptosis (PRIMA), PRIMA-1^Met^; V + P: vemurafenib + PRIMA-1^Met^. * *p* < 0.05; ** *p* < 0.01; *** *p* < 0.001 (Student’s *t*-test) compared to RT alone. (**C**) The interaction between Vemu, PRIMA, and RT was examined using the combination index (CI) method of Chou and Talalay and CompuSyn software. CI = 1, additive effect, CI <1, synergism, CI >1, antagonism. (**D**) Cell death (apoptosis (annexin-V positive cells) + necrosis (7-AAD positive cells) analysis for irradiated and non-irradiated cells treated with 1 µM of vemurafenib and/or 40 µM of PRIMA-1^Met^. Data are presented as means ± SD (*n* = 3) compared to non-irradiated cells, *** *p* < 0.001 (Student’s *t*-test). SD, standard deviation.

In order to evaluate the benefit of combining RT with p53 activation and MAPK inhibition, we designed a workflow where one day after drug exposure, melanoma cells were irradiated with one single dose of 2 Gy, 5 Gy, or 10 Gy. Protein analysis was done one day after irradiation, in order to assess the direct effect of irradiation and effectors (vemurafenib and PRIMA-1^Met^). Cell death and colony formation were done one and two weeks later, respectively, to evaluate the biological effect of irradiation (Figure 1A). Fresh medium with the effectors was added every three days. First, we investigated the effect of vemurafenib and/or PRIMA-1^Met^ on the radiosensitivity of melanoma cells using clonogenic survival analysis. It is of note that both vemurafenib and PRIMA-1^Met^ were used at fixed effective (on targets) and non-toxic (for cells) concentrations. Irradiation with 2 Gy, 5 Gy, and 10 Gy (Figure 1B) significantly decreased cell survival in a dose-dependent manner. The addition of the BRAF inhibitor or the p53 activator before RT treatment significantly reduced clonogenic survival in both cell lines with intrinsic (MM043) and acquired resistance (MM074-R) to vemurafenib. Interestingly, the combination index (Figure 1C) shows that combining p53 activation + BRAF inhibition + irradiation had an additive effect (0.8 < CI ≤ 1) with 2 Gy, a synergistic effect (CI ≤ 0.6) with 5 Gy, and a slight additive and antagonistic effect (CI ≥ 1.1) with 10 Gy. Of note, 10 Gy was already cytotoxic alone, explaining the lack of synergy of this dose in combinations.

More importantly, combining vemurafenib and PRIMA-1^Met^ potentiates the RT effect in melanoma cells and induces the better inhibition of cell survival and a massive induction of cell death compared to the effect of irradiation and p53 reactivation or irradiation and BRAF inhibition (Figure 1D). 

Although several data about tumor cell death induction by RT and/or chemotherapeutic agents were published in recent years, the focus was mainly set on high single doses of X-rays [44]. In other studies, it was suggested that ionizing radiations delivered in a fractionated regime amplify the survival advantage of normal tissues over cancer cells, largely based on the better sublethal damage repair of radiation damage in normal cells as compared to cancer cells. Most RT regimes now consist of daily fractions of 1.5 to 3 Gy, which are given during several weeks [45]. To get hints on how clinically relevant treatment schemes influence the cell survival of melanoma cells, and to find the optimal radiation therapy regime, we conducted an assay about colony formation after fractionated RT on two consecutive days with 2 × 2.5 Gy and 2 × 5 Gy in combination with clinically relevant concentrations of vemurafenib and/or PRIMA-1^Met^. Supplementary Figure S1 displays cell survival forms of MM043 and MM074-R melanoma cells 12 days after the last treatment with fractionated RT, vemurafenib, PRIMA-1^Met^ alone, or their combinations. In both cell lines, fractionated RT schedules with 2 × 2.5 Gy and 2 × 5 Gy are less cytotoxic than single doses of 5 Gy and 10 Gy, respectively. However, 2 × 5 Gy and 10 Gy alone are very cytotoxic and not suitable for combination. However, in both cell lines, the best synergistic effect was observed by combining vemurafenib (CI = 0.21), PRIMA-1^Met^ (CI = 0.33), and a single RT dose of 5 Gy.

### 2.2. Activation of Both MAPK and PI3K/AKT Pathways Are a Frequent Event in Melanoma Radioresistance

**Figure 2:**
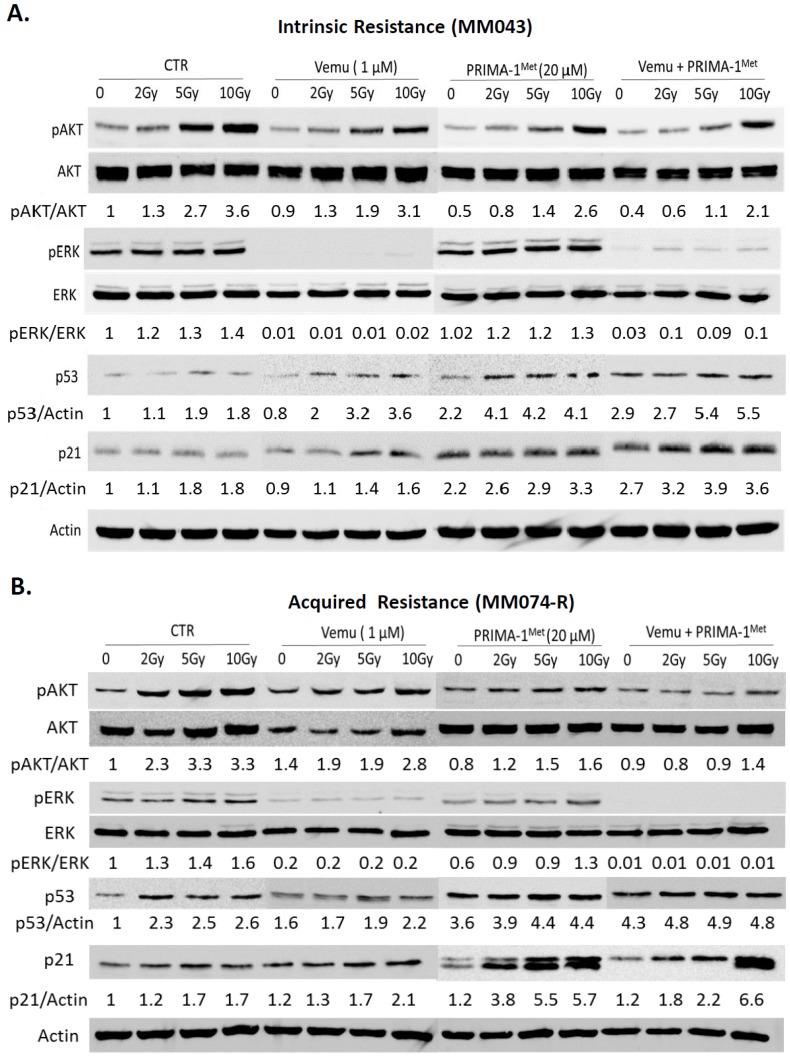
Mitogen-activated protein kinase (MAPK) kinase and PI3K pathways stimulation confers resistance to radiotherapy (RT) in BRAF mutant melanoma cells in vitro. Effect of RT with 2 Gy, 5 Gy, and 10 Gy in combination with 1 µM of vemurafenib and/or 20 µM of PRIMA-1^Met^ on the PI3K/AKT, MAPK, and p53/p21 pathways. (**A**) Cells with intrinsic resistance to vemurafenib (MM043) and (**B**) MM074-R with acquired resistance to vemurafenib. β-actin was used as loading control.

To understand the observed results and confirm target activation after irradiation, we evaluated the effect of these combinations on the p53, MAPK, and PI3K (Phosphoinositide 3-kinases) pathways by Western blot (Figure 2). We found that RT alone is associated with an important activation of ERK (Extracellular signal regulated kinase) and AKT in a dose-dependent manner in both cell lines. As expected, RT increases p53 expression and activity, and is reflected by the expression of p21. Combining a BRAF inhibitor and RT shows vemurafenib to completely oppose pERK induction by RT. In both cell lines, combining p53 activation and RT induces p53 and p21WAF to levels greater than either treatment or RT alone and opposes pAKT upregulation. Importantly, combining BRAF inhibition, p53 activation, and RT reduced pAKT as well as a single treatment of vemurafenib and PRIMA-^Met^, while ERK phosphorylation is more affected by the combination with an important activation of p53 and p21 in cells with intrinsic (Figure 2A) and acquired resistance (Figure 2B) to vemurafenib.

### 2.3. Evaluation of the Radiosensitizing Effect of Reactivating p53 under BRAF Inhibition In Vivo 

**Figure 3:**
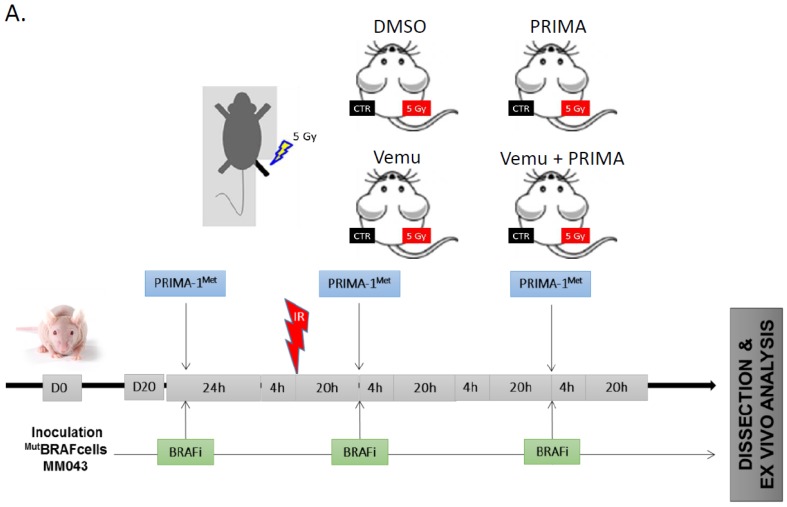

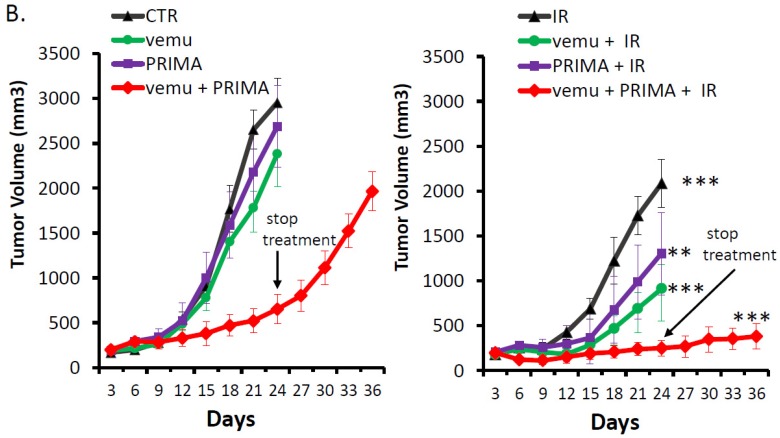
p53 activator (PRIMA-1^Met^) and BRAF inhibitor (vemurafenib) radiosensitise ^V600E^BRAF mutant melanoma in vivo. (**A**) Swiss nude mice (nu/nu) were injected by either MM043 and treated with CTR (DMSO), vemurafenib (45 mg/kg), PRIMA-1^Met^ (50 mg/kg), or vemurafenib and PRIMA-1^Met^. One day later, the mice were irradiated on the right leg. Data are presented as means ± SEM (*n* = 9) compared to untreated tumors (**B**) Tumor volume of mice, non-irradiated (left panel), irradiated (right panel), untreated or treated with vemurafenib and/or PRIMA-1^Met^ in the period between D0 and D36. ** *p* < 0.01, *** *p* < 0.001 (two-way ANOVA) compared to non-irradiated.

Our next aim was to validate the obtained results in an animal model (Figure 3). The radiosensitivity of MM043 melanoma cells after BRAF inhibitors and p53 reactivation was investigated. After similar subcutaneous tumor cell injection on each leg, tumor growth was monitored to reach volumes of about 200 mm^3^ before intraperitoneal administration of the effectors (i.e., 45 mg/kg vemurafenib and/or 50 mg/kg PRIMA-1^Met^). Vemurafenib and PRIMA-1^Met^ were used in a concentration that individually did not cause any major inhibition of tumor growth. On day 1 after initial treatment, the mice were subjected to single dose RT (5 Gy) of the right leg, while protecting the rest of the body with a lead shield (Figure 3A). RT alone slightly decreased tumor growth compared to that which was non-irradiated (left panel, untreated control, or CTR) but importantly, the combination of BRAF inhibition plus RT or RT plus p53 activation showed a synergistic effect on tumor growth inhibition (CI = 0.2 and 0.68, respectively) in comparison with non-irradiated tumors (left panel). Interestingly, when RT was added to the combination of both effectors, the tendency showed an even better tumor control (Figure 3B). In a next step, we investigated the effect of this combination +/− RT on the residual cells (which can be the cause of future acquired resistance). For this purpose, we stopped the drug treatment on day 24 and saw the residual cells to resume growth in an exponential manner only in non-irradiated tumors (Figure 3B). 

**Figure 4:**
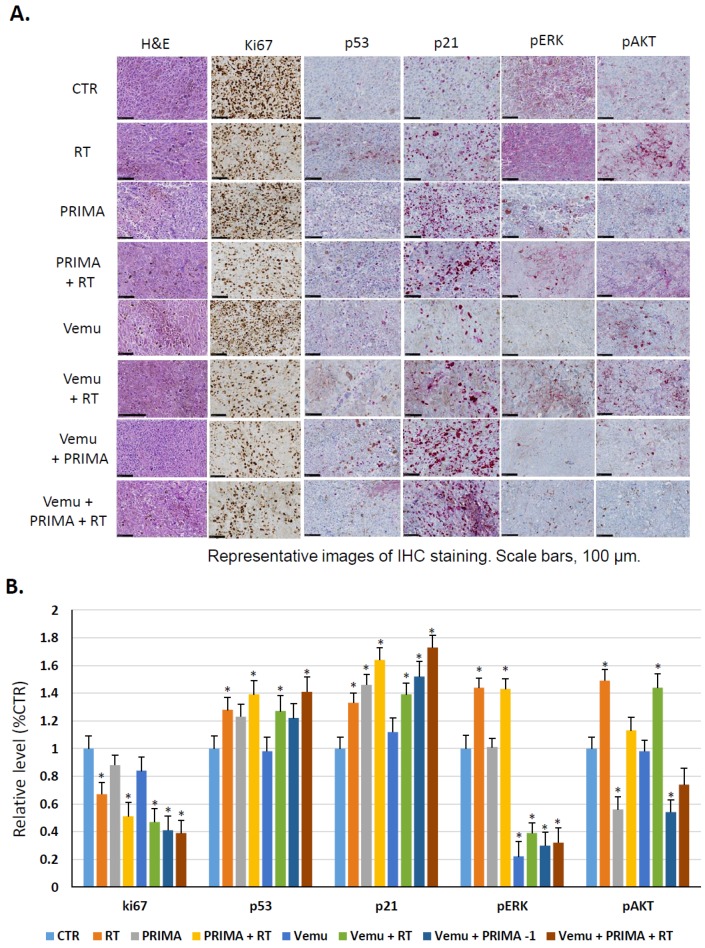
MAPK kinase and PI3K pathways reactivation confers resistance of BRAF mutant melanoma cells to radiation in a nude mice model. (**A**) Immunohistochemistry staining for Ki67, p53, p21, pERK, and pAKT in non-irradiated cells and irradiated cells, untreated or treated with vemurafenib (45 mg/kg) and/or PRIMA-1^Met^ (50 mg/kg). (**B**) Relative expression of immunohistochemistry data reporting Ki67, p53, p21, pERK, and pAKT compared to control. Data are presented as means ± SEM from three mice compared to untreated tumors (CTR). The level of significance is indicated by * *p* < 0.05 (Student’s *t*-test).

Following this confirmation of the triplet therapy, we investigated the effect of RT alone as well as in combination with vemurafenib and/or PRIMA-1Met on the diverse proteins that are crucial in the MAPK, PI3K, and p53 signaling pathways. IHC analysis (Figure 4) demonstrated that RT alone (5 Gy) is associated with a slight decrease of Ki67 positive cells, but with a significant upregulation of p53, p21, pERK, and pAKT. Interestingly, p53 reactivation and RT together induce the significant activation of p53 and consequently p21 upregulation while antagonizing the activation of AKT compared to tumors treated with RT alone. On the other hand, the combination of vemurafenib with RT results in a complete inhibition of ERK phosphorylation in comparison with exposure to RT alone. Thus, BRAF inhibition, p53 reactivation, and RT together reduce proliferation, as evidenced by Ki67 decrease, an increase of p53 and p21 protein expression, and the inhibition of both MAPK and PI3K/AKT.

These data strongly suggest that the activation of both MAPK and PI3K/AKT pathways are a frequent event in melanoma radioresistance, and combining PRIMA-1^Met^ with Vemurafenib acts synergistically with RT to trigger melanoma growth inhibition by affecting these two important signaling pathways.

## 3. Discussion

There is no universally accepted definition of radioresistant melanoma. However, the surviving fraction at 2 Gy (SF2) is a common measure of intrinsic radiosensitivity; SF2 ≥0.5 being considered radioresistant is a widely used criteria [46]. Recent reports show variable radiosensitivity in melanoma patients and established melanoma cell lines (SF from 2.2 to 8 Gy) [47]. There has also been demonstrations of differential radiosensitivity based upon mutation profiles. Moreover, new RT techniques such as stereotactic body RT (SBRT) and combinations with systemic treatments are proposed to overcome radioresistance. SBRT delivers high doses per fraction (≥6 Gy), inducing more immediate cell death while stimulating the immune system. Increasing evidence indicates that radiotherapy recruits biological effectors outside the treatment field and has systemic effects: the so-called abscopal effect [48]. In addition, patients treated with SBRT and various systemic immunologic and targeted melanoma agents showed a significant difference in the overall survival of patients with distant melanoma brain metastasis [49]. 

Despite melanoma radioresistance, irradiation is indicated in advanced regional disease in order to improve regional control. Therefore, we investigated the molecular targets that could be used to ameliorate and maximize the benefit from irradiation, particularly in BRAF mutant melanoma. Recently, the radiosensitizing effect of both BRAF inhibitors vemurafenib and dabrafenib has been described [50,51,52,53]. Using melanoma cells with intrinsic and acquired resistance to vemurafenib, we demonstrate here the combination of radiotherapy and a BRAF inhibitor to cause a radiation dose-dependent decrease in cell survival, and consequently an increase in cell death in vitro. We can conclude that there is a clear radiosensitizing effect of BRAF inhibitors in BRAF-mutated melanoma cells, and these data are supported by other reports [23,54]. Consequently, the antitumor effect provided by both radiotherapy and BRAF inhibitors could be enhanced when used together. This is of particular importance in patients with multiple brain metastases, regarding their poor prognosis [23]. Our data are in concordance with previous studies [54,55], but recently, Walter et al. [56] seems to demonstrate that BRAF inhibitors do not act synergistically with radiation. However, they did not include the role of p53, and the calculation of survival was conducted too shortly (24 h) after radiation exposure, while it is well known that one needs to wait at least four or five population doubling times before performing cell line survival experiments [57]. In another study on melanoma cells, combining cytotoxic drugs (temozolomide, carboplatin, paclitaxel, and vemurafenib) and RT showed the best combination index with 5 Gy [47]; these data were validated in our study. 

One of the key molecules involved in a cell’s response to RT is p53. Indeed, p53 is described as the central regulator of the checkpoint activated by DNA damage. Low-dose irradiation is thought to induce low-level DNA damage and a reversible cell-cycle arrest, whereas higher dose irradiation triggers apoptosis [28,58]. Therefore, active wild-type p53 is thought to sensitize tumors more to RT through cell death induction, and its inactivation contributes to treatment resistance [59,60]. Therefore, p53 is considered as a pharmacological target to increase the efficacy of cancer treatment [61] and sensitivity to radiotherapy [62,63]. 

In cutaneous melanoma, p53 is mutated in only 17%, but its activity is attenuated in 90% due to the overexpression of its negative regulators MDM4, MDM2, and iASPP [39,64]. Importantly, our previous work [36] showed that PRIMA-1^Met^ reactivates p53 regardless of the mechanism causing its deactivation, thereby inhibiting PI3K signaling and sensitizing (V600E/K) BRAF-positive melanoma cells to BRAF inhibitors. In the present study, we demonstrated that the radiation effect on cell death was reinforced when used in combination with a BRAF inhibitor and/or p53 reactivation in both melanoma cells with intrinsic and acquired resistance to BRAF inhibition.

Several studies indicate the PI3K/AKT pathway to be implicated in all the major mechanisms of radioresistance [65,66,67,68]. Ionizing irradiation produces double-strand and single-strand DNA breaks followed by an accumulation of functionally active p53 protein. One pathway downstream of p53 after irradiation is mediated through p21, which is a general cyclin-dependent kinase inhibitor [69]. In this study, we found that the irradiation of melanoma cells is associated with increases in pAKT, p53, and p21. Consequently, the treatment of melanoma cells with radiation and PRIMA-1^Met^ increases p53 levels and decreases AKT phosphorylation [36]. Indeed, active p53 is known to stimulate PTEN (phosphatase and tensin homolog) and negatively regulate the p110 catalytic subunit of PI3K, leading to the strong inhibition of pAKT [70,71]. 

Thus, altogether, direct MAPK inhibition by vemurafenib, indirect PI3K/AKT downregulation by PRIMA-1^Met^ [36], and the supplemental effect by RT and PRIMA-1^Met^ on p53 activation may explain the observed synergistic effect of the triplet BRAF inhibitor, p53 reactivator, and RT on BRAF mutant melanoma cells both in vitro and in vivo. 

The rational design of potent sensitizers for use in cancer radiotherapy has become an important strategy for the discovery of targeted therapies for personalized cancer medicine. However, Hecht et al. found that RT with concomitant BRAF inhibitor therapy is feasible with an acceptable increase in toxicity, and that vemurafenib is a more potent radiosensitizer than dabrafenib [72]. Moreover, the interruption of vemurafenib treatment during radiation was associated with better survival and less toxicity compared to continuous concomitant treatment [11]. In addition, active p53 accumulates in the nucleus and causes a high responsiveness of p53 in chronic IR-treated breast cancer cells [73]. In this study, we started the combination of BRAF inhibition and p53 activation one day before irradiation with the aim of avoiding radioresistance on one hand by the inhibition of both MAPK and PI3K pathways, and increasing radiosensitivity by p53 activation on the other hand. 

This radiosensitization is also pertinent for subsequent immunotherapy protocols with checkpoint inhibitors, as RT plus radiosensitizers followed by immunotherapy could enhance the immune response even more.

Alltogether, there is compelling rationale to identify promising molecular targeting agents that may sensitize tumor cells to RT. In this preclinical study, we showed that p53 activation significantly improves the radiosensitizing effect of BRAF inhibition in ^V600E^BRAF mutant melanoma. 

## 4. Materials and Methods

### 4.1. Inhibitors

The ^V600E^BRAF inhibitor vemurafenib (PLX4032) (Nuclilab, Ede, The Netherlands) and the p53 activator PRIMA-1^Met^ (aprea therapeutics) (Karolinska Institutet Science Park, Solna, Sweden) were dissolved according to the manufacturer’s recommendation at 10^−2^ M; then, they were aliquoted and stored at −20 °C.

### 4.2. Melanoma Cell Lines

Mutated BRAF human melanoma cell lines with intrinsic (MM043) and acquired resistance (MM074-R) to vemurafenib used in this study were established in our laboratory and described previously [36]. The BRAF, NRAS, TP53, and PTEN mutation status have been evaluated with the next-generation DNA sequencing for 48 genes from cancer panel (TruSeq Amplicon—Cancer Panel, Illumina, San Diego, CA, USA) [20,32,36]. 

### 4.3. Cell Culture Conditions

Cells were grown in HAM-F10 medium supplemented with 5% heat-inactivated fetal calf serum, 5% heat-inactivated new-born calf serum, and 200 mL of L-glutamine, 100 U/mL of penicillin, and 100 ug/mL of streptomycin at standard concentrations (all from Gibco, Invitrogen, UK) (culture medium) at 37 °C in a humidified 95% air and 5% CO_2_ atmosphere. For routine maintenance, cells were propagated in flasks, harvested by trypsinization (0.05% trypsin-EDTA) (Gibco) and subcultured twice weekly. Cell count and volume were evaluated using a TC10™ Automated Cell Counter (Bio-Rad, Hercules, CA, USA). All the cell lines were regularly checked for mycoplasma contamination using MycoAlert^®^ Mycoplasma Detection Kit (Lonza, Rockland, ME, USA).

### 4.4. Clonogenic Assay

Cell survival was measured by clonogenic assay, according to Franken et al. [57]. Briefly, on day 0, between 150–20,000 cells, depending on the treatment, were seeded in triplicate in six-well plates and incubated under regular culture conditions. On day 1, cells were treated with vemurafenib, PRIMA-1^Met^, or both, and subsequently incubated under regular culture conditions. On day 2, cells were irradiated. Cells were irradiated in a 6-MV beam from a Clinac 600 linear accelerator (Varian Medical Systems, Palo Alto, CA, USA). The collimator opening was set to 40 × 40 cm^2^, which gives the possibility of irradiating several plates in one batch. In order to achieve a good dose homogeneity and electronic equilibrium, a 6-mm thick polystyrene build-up was put on top of the plates. Plates were placed on a 5-cm thick polystyrene phantom for adequate backscattering conditions. The dose rate was set to 4 Gy per minute, so that the full experiment took less than 20 minutes and can be easily accommodated in a clinically used linear accelerator. On day 14, cells were fixed and stained with 6% glutaraldehyde and 0.05% crystal violet. Colonies of at least 50 cells were counted. The survival fraction was calculated either relative to untreated samples.

### 4.5. Western Blot Analysis

Cells were plated in Petri dishes (3 × 10^6^ cells/dish) in culture medium. One day after plating, the culture medium was replaced by a fresh medium with or without effectors. One day later, cells were irradiated and incubated for 30 min or 24 h [74]. At the end of cell exposure to drugs, cells were lysed using a detergent cocktail (M-PER Mammalian Extraction Buffer) supplemented with protease inhibitors (Halt Protease Inhibitor Cocktail) and phosphatase inhibitors (Halt Phosphatase Inhibitor Cocktail) (all from Thermo Fischer Scientific, Waltham, MA USA). Protein concentrations were determined by the BCA Protein Assay (Thermo Fischer Scientific) using bovine serum albumin as the standard. Equal amounts of extracted proteins (35 µg) were subjected to 10% or 12% SDS-PAGE and electrotransferred onto nitrocellulose membranes using an iBlot^®^ Dry Blotting System (Invitrogen, Life Technologies, Gent, Belgium). Immunodetections were performed using primary antibodies. Immunodetections were performed using antibodies raised against pAKT (Ser 473) (D9E, 1/500), AKT (40D4, 1/1000), p21 (Waf1/Cip1) (12D1, 1/1000) (all from Cell Signaling Technology, Danvers, MA, USA), pERK (Tyr 204) (E-4, 1/1000), ERK2 (C-14, 1/2000), p53 (DO-1, 1/200) (all from Santa Cruz Biotechnology, Santa Cruz, CA, USA), and β-actin (C4, 1/5000) (from Millipore, Temecula, CA, USA). Peroxidase-labeled anti-rabbit IgG antibody (1/5000) or peroxidase-labeled anti-mouse IgG antibody (1/5000) (both from Amersham Pharmacia Biotech, Roosendaal, The Netherlands) were used as secondary reagents to detect corresponding primary antibodies. Bound peroxidase activity was revealed using the SuperSignal^®^ West Pico Chemiluminescent Substrate (Thermo Fischer Scientific) following the manufacturer’s indications. Immunostaining signals were digitalized with a PC-driven LAS-3000 charge-coupled device (CCD) camera (Fujifilm, Tokyo, Japan), using a software specifically designed for image acquisition (Image Reader, Raytest^®^, Straubenhardt, Germany). Immunoreactive band intensities were quantified using the software AIDA^®^ Image Analyser 3.45 (Raytest^®^) (Figure S2).

### 4.6. Cell Death Determination

Cells were seeded in six-well plates (2 × 10^5^ cells/well) in culture medium. One day after plating, the culture medium was replaced by a fresh one containing effectors or not. Cells were irradiated one day later and incubated for six additional days. Then, the supernatant was collected and adherent cells were harvested by trypsinization and returned back to the previously collected medium. Cells were pelleted by brief centrifugation (200× *g*, 5 min) and suspended in 100 µL of 1× Binding Buffer (BD Pharmingen, Franklin Lakes, NJ, USA). After the addition of 5 µL of annexin V-PE and 5 µL of 7-amino-actinomycin (7-AAD), cell suspensions were incubated for 15 min at room temperature and in the dark. Finally, cells were diluted with 400 µL of Binding Buffer and analyzed within one hour in a flow cytometer (FACS Calibur, Becton Dickinson, Franklin Lakes, NJ, USA).

### 4.7. Human Melanoma Xenografts

Five to six-week-old female nude (nu/nu) mice weighing 17–21 g were purchased from Charles River Laboratories (Saint Aubin lès Elbeuf, France). Mice were injected subcutaneously (right and left leg) with 5 × 10^6^ MM043 cells (with intrinsic resistance to vemurafenib) in 150 µL of 50% Matrigel (from Trevigen, Gaithersburg, MD, USA) in saline solution. When tumors reached ~200 mm^3^, mice were randomized into four groups of nine mice and daily intraperitoneally injected with vehicle (DMSO), 45 mg/kg of vemurafenib and/or 50 mg/kg of PRIMA-1^Met^ (Figure 3A). One day after the first treatment, mice were irradiated in an X-RAD 320 Micro-irradiator (Precision X-Ray Irradiation, North Branford, CT, USA). Irradiation was restricted to the right leg, the rest of the body was protected by a lead shield. Tumor size and body weight were measured every three days. Tumor volumes were calculated using the formula (L × W × W)/2 [20], in which L is the length and W is the width, as measured with a Vernier caliper. Immediately after dissection, tumors xenografts were fixed and embedded in paraffin. The experiments were performed in accordance with the European Union Guidelines and validated by the local Animal Ethics Evaluation Committee “Comité d’éthique du Bien-Etre Animal-Université Libre de Bruxelles” (CEBEA) protocol: 692N.

### 4.8. Immunohistochemistry Staining 

After dissection, tumors were immediately fixed in 10% formalin until the ex vivo study was performed. Then, samples were transferred to 70% ethanol and stored at 4 °C. Samples were processed and embedded in paraffin, and 4-μm sections were prepared for immunostaining with hematoxylin and eosin (HE), Ki67 (MIB-1, 1/50, Thermo Fisher Scientific, Inc., Waltham, MA, USA), pAKT (Ser 473, 1/100), p21 (Waf1/Cip1, 1/50) (both from Cell Signaling Technology), pERK (Tyr 204, E-4, 1/100), and p53 (DO-1, 1/50) (both from Santa Cruz). Stained sections were imaged using an NDP Slice Scanner (Hamamatsu, Hamamatsu City, Japan). Three regions were selected at random on different parts of the section and analyzed at ×15 magnification, using ImmunoMembrane and ImmunoRatio web applications.

### 4.9. Statistical Analysis

Survival fraction, cell death, and protein expression level data were expressed as means ± SD of at least three independent experiments; statistical significance was measured by Student’s *t*-test using GraphPad Prism software. Differences in tumor volumes among groups of treated mice were tested using two-way ANOVA; values are presented as means ± SEM.

### 4.10. Combination Index Calculation

The synergistic effect was analyzed by the multiple drug-effect equation and quantified by the combination index (CI) using CalcuSyn software version 2.1 (Biosoft, Cambridge, UK). CI values between 0.9–1.1 indicates an additive effect; values between 0.7–0.9 indicated a moderate synergism; values lower than 0.7 indicated a strong synergism; and antagonism is represented by CI values higher than 1.1.

## 5. Conclusions

RT is rather used in palliative settings in melanoma patients, as such disease is considered to be radioresistant. Interestingly, MAPK inhibition or p53 activation are both reported as radiosensitizers, but their combination has not been tested in melanoma yet. In this study, we provide evidence that combining MAPK inhibition to p53 reactivation significantly enhances the radiosensitivity of melanoma both in vitro and in vivo. Therefore, there is a compelling rational to use radiotherapy in curative settings in melanoma patients under MAPKi and p53 activator combination.

## Supplementary Materials

The following are available online at https://www.mdpi.com/2072-6694/11/8/1093/s1, Figure S1: Difference in single and fractionated dose effect of radiation, p53 activation and BRAF inhibition on BRAF mutant melanoma cells. A. Effectors were added one day before irradiation. To evaluate the biological effect of irradiation, colony formation was evaluated after two weeks. Effectors with fresh medium were changed every 3 days. B. Clonogenic survival assay of human melanoma cell lines with intrinsic resistance (MM043) and acquired resistance (MM074-R) to vemurafenib 12 days after irradiation with 2.5, 5 and 10 Gy or 2 × 2.5 and 2 × 5 Gy alone or in combination with vemurafenib (Vemu, 0.1 µM) and/or PRIMA-1^Met^ (PRIMA-1^Met^, 20 µM). C. Surviving fractions were calculated relative to plating efficiencies. Data were presented as mean ± standard error of at least 3 independent experiments. Gy, Gray; CTR: untreated control; Vemu, vemurafenib; PRIMA, PRIMA-1^Met^; V+P: vemurafenib + PRIMA-1^Met^, Figure S2: The whole blot showing all the bands on the Western.
