# Correction: Krayem et al. The Benefit of Reactivating p53 under MAPK Inhibition on the Efficacy of Radiotherapy in Melanoma. *Cancers* 2019, *11*, 1093

**DOI:** 10.3390/cancers15245860

**Published:** 2023-12-15

**Authors:** Mohammad Krayem, Malak Sabbah, Ahmad Najem, An Wouters, Filip Lardon, Stephane Simon, François Sales, Fabrice Journe, Ahmad Awada, Ghanem E. Ghanem, Dirk Van Gestel

**Affiliations:** 1 Laboratory of Oncology and Experimental Surgery, Institut Jules Bordet, Université Libre de Bruxelles, Rue Héger-Bordet 1, 1000 Brussels, Belgiumfabrice.journe@bordet.be (F.J.);; 2 Department of Radiation Oncology, Institut Jules Bordet, Université libre de Bruxelles, 1000 Brussels, Belgium; 3 Center for Oncological Research (CORE), University of Antwerp, 2610 Wilrijk, Belgiumfilip.lardon@uantwerpen.be (F.L.); 4 Department of Human Anatomy and Experimental Oncology, Université de Mons (UMons), Research Institute for Health Sciences and Technology, 7000 Mons, Belgium; 5 Department of Internal Medicine, Institut Jules Bordet, Université Libre de Bruxelles, 1000 Brussels, Belgium

In the original article [1], there was a mistake in Figure 2A as published. The actin blot in our publication in *Cancers* (Figure 2A) had already been used in our published paper in Oncotarget in 2018. This is probably due to an error in our image saving. Note that this mistake does not change or alter the content and conclusions of our paper. The mistake indeed concerns control experiments, which were very easily reproduced. The corrected Figure 2A appears below.


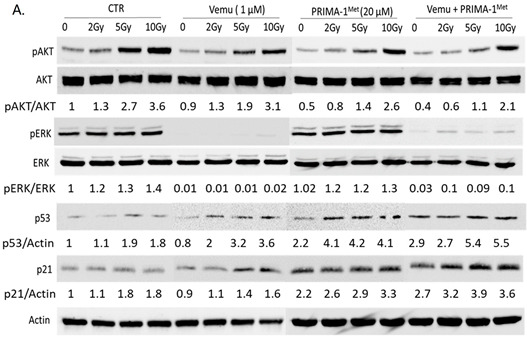


In Figure 2B’s P53 panel, there are vertical splices between lanes 4/5 and 12/13. There are no apparent splices between these lanes in the other panels. This is because samples for every group (i.e., four conditions) were conducted on different membranes.

The authors state that the scientific conclusions are unaffected. This correction was approved by the Academic Editor. The original publication has also been updated.

## References

[1] Krayem M., Sabbah M., Najem A., Wouters A., Lardon F., Simon S., Sales F., Journe F., Awada A., Ghanem G.E. (2019). The Benefit of Reactivating p53 under MAPK Inhibition on the Efficacy of Radiotherapy in Melanoma. Cancers.

