# Supplementary material for: The Benefit of Reactivating p53 under MAPK Inhibition on the Efficacy of Radiotherapy in Melanoma
Source: Cancers (Basel). 2019 Aug 1;11(8):1093. doi: 10.3390/cancers11081093 (PMC6721382; doi:10.3390/cancers11081093)
Supplement: Supplementary file 1 [file cancers-11-01093-s001.pdf]

Supplementary Material: The Benefit of Reactivating p53 under MAPK Inhibition on the Efficacy of Radiotherapy in Melanoma

Mohammad Krayem, Malak Sabbah, Ahmad Najem, An Wouters, Filip Lardon, Stephane Simon, François Sales, Fabrice Journe, Ahmad Awada, Ghanem E. Ghanem and Dirk Van Gestel

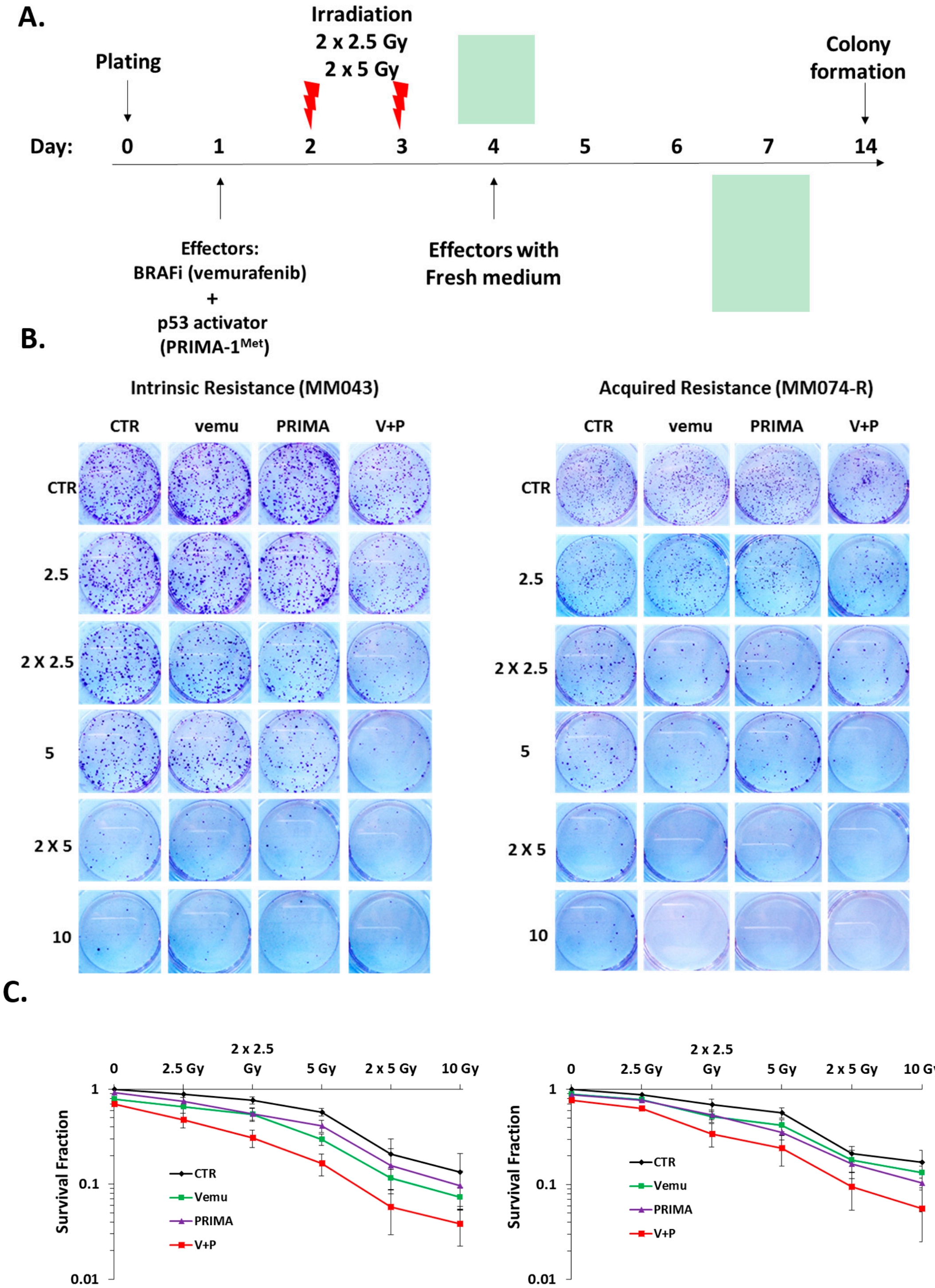

Figure S1. Difference in single and fractionated dose effect of radiation, p53 activation and BRAF inhibition on BRAF mutant melanoma cells. (**A**) Effectors were added one day before irradiation. To

evaluate the biological effect of irradiation, colony formation was evaluated after two weeks. Effectors with fresh medium were changed every 3 days. (**B**) Clonogenic survival assay of human melanoma cell lines with intrinsic resistance (MM043) and acquired resistance (MM074-R) to vemurafenib 12 days after irradiation with 2.5, 5 and 10 Gy or 2 × 2.5 and 2 × 5 Gy alone or in combination with vemurafenib (Vemu, 0.1 μM) and/or PRIMA-1^{Met} (PRIMA-1^{Met}, 20 μM). (**C**) Surviving fractions were calculated relative to plating efficiencies. Data were presented as mean ± standard error of at least 3 independent experiments. Gy, Gray; CTR: untreated control; Vemu, vemurafenib; PRIMA, PRIMA-1^{Met}; V+P: vemurafenib + PRIMA-1^{Met}.

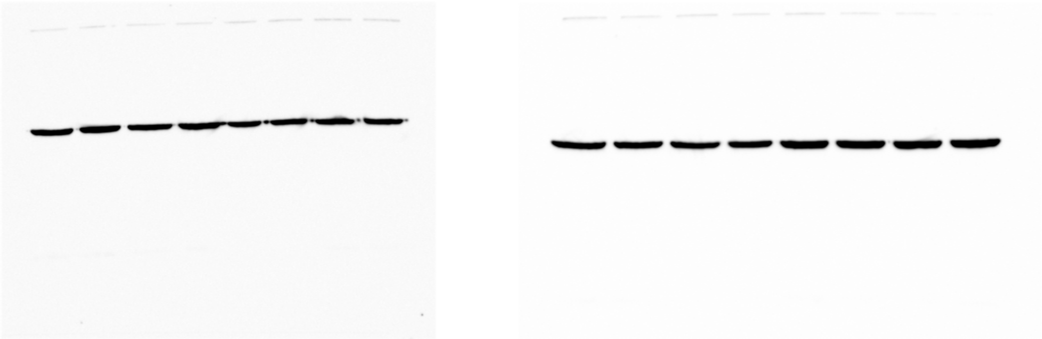

Actin

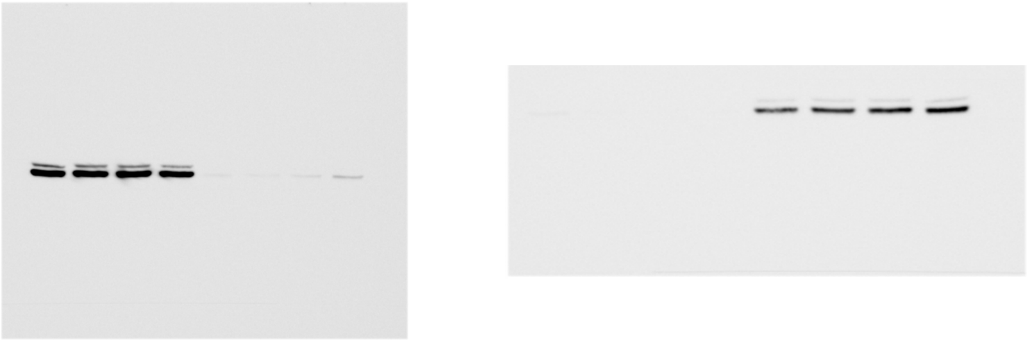

pERK

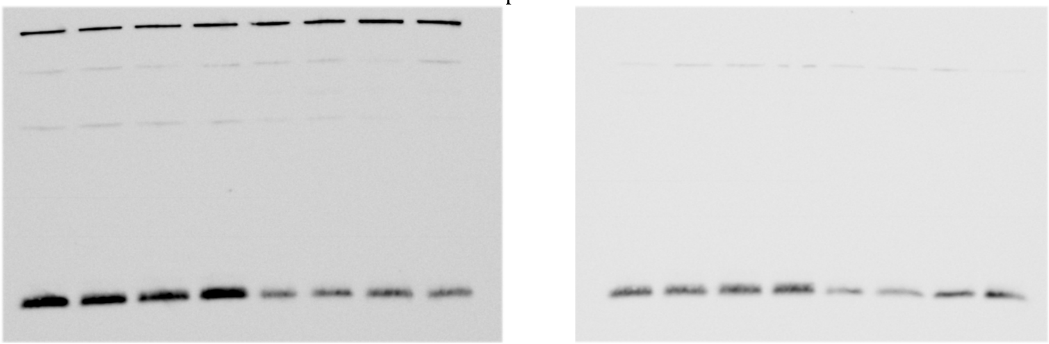

P12

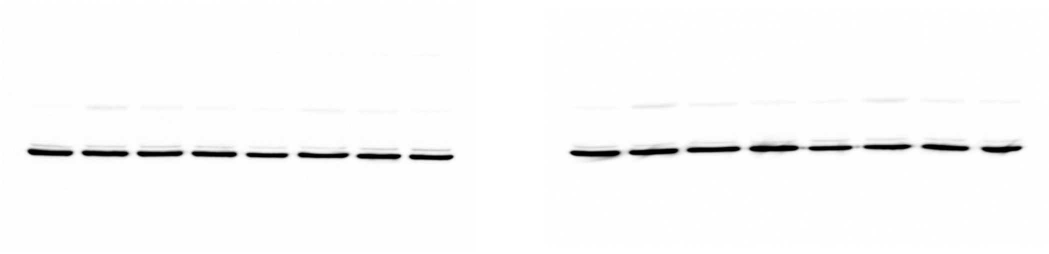

ERK

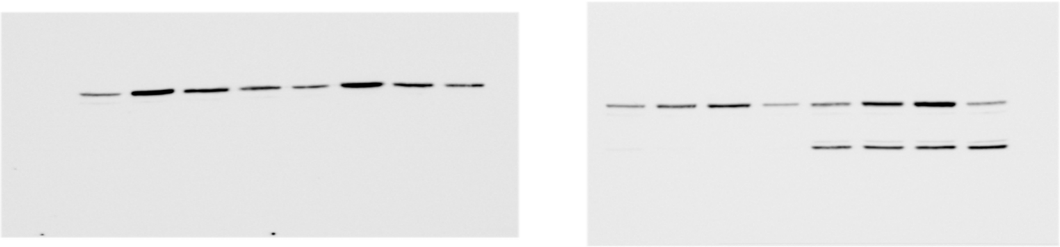

pAKT

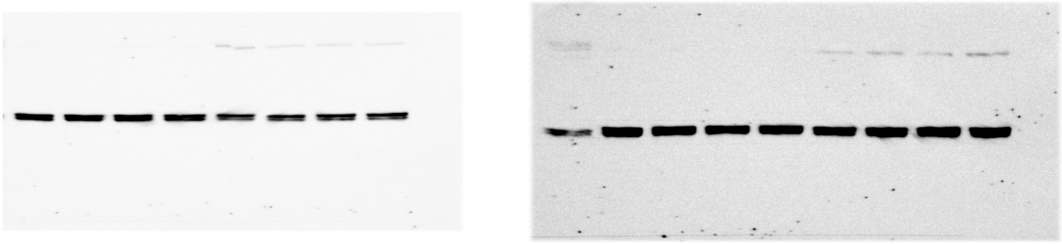

AKT

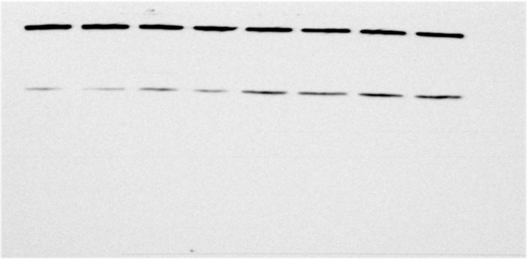

P53

Figure S2. The whole blot showing all the bands on the Western.

© 2019 by the authors. Licensee MDPI, Basel, Switzerland. This article is an open access article distributed under the terms and conditions of the Creative Commons Attribution (CC BY) license (http://creativecommons.org/licenses/by/4.0/).
